# Student Perceptions and Barriers Related to Developing Tactile Knowledge of Removable Prostheses: A Questionnaire-Based Study in Professional Institutes

**DOI:** 10.7759/cureus.98300

**Published:** 2025-12-02

**Authors:** Anshul Chugh, Mariko Hattori, Neha Sikka, Adarsh Kumar, Noriyuki Wakabayashi

**Affiliations:** 1 Prosthodontics/Maxillofacial Prosthetics, Pandit Bhagwat Dayal Sharma University of Health Sciences, Rohtak, IND; 2 Advanced Prosthodontics, Institute of Science, Tokyo, JPN; 3 Dental Materials, Postgraduate Institute of Dental Sciences, Pandit Bhagwat Dayal Sharma University of Health Sciences, Rohtak, IND; 4 Public Health Dentistry, Postgraduate Institute of Dental Sciences, Pandit Bhagwat Dayal Sharma University of Health Sciences, Rohtak, IND

**Keywords:** barriers, clinical knowledge, questionnaire study, removable prosthesis, student teaching

## Abstract

Background and objective

Building proficiency in removable prosthodontics (RP) involves significant challenges for undergraduate dental students due to complexities associated with understanding threshold concepts and internalizing tactile knowledge. This study primarily aimed to systematically analyze the barriers that hinder undergraduate dental students’ understanding of threshold concepts and the development of tactile knowledge required to achieve proficiency in RP. Additionally, it sought to identify essential knowledge and skills necessary for competency in RP among third- to fifth-year dental students, to evaluate differences in students’ knowledge, skills, and clinical performance across different clinical years, and to propose strategies - including improved clinical exposure, hands-on practice, and mentorship - to help students overcome identified barriers and advance their proficiency in RP.

Methods

This study involved third-, fourth-, and fifth-year dental students (N = 105, 203, and 69, respectively). A self-administered questionnaire was distributed in both online and offline formats. The collected data were analyzed using descriptive statistics.

Results

Analysis of gender differences revealed that female participants significantly outnumbered male participants, and female students delivered more complete dentures (F = 256, M = 121). When comparing clinical years, significant differences were identified in the knowledge of procedural steps, anatomical structures, and readiness for clinical tasks (p<0.05). The number of complete dentures delivered varied significantly between third- and fifth-year students, as well as between fourth- and fifth-year students (p<0.05), highlighting a progressive improvement in skills with clinical experience.

Conclusions

Overcoming the barriers highlighted in this study requires a multifaceted approach involving enriched clinical exposure, extended hands-on practice, and enhanced mentorship to enable students to attain the necessary proficiency in RP.

## Introduction

Removable dental prostheses play a vital role in restoring smiles, improving functional performance, and enhancing overall well-being [1]. Competency extends beyond knowledge, encompassing a blend of skills, professional attitude, and ethical practice, which together lay the foundation for a successful dental career. For dental students, acquiring core competencies during their undergraduate years is crucial for confidently delivering high-quality removable denture services. Mastering these essentials enables students to progress from a beginner to a skilled professional, preparing them for real-world challenges and independent practice [2,3]. This represents a critical stage in the development of expertise along the novice-expert continuum, which includes stages ranging from novice to expert, with intermediate phases such as beginner, competent, and proficient [2,4].

In the beginner stage, learners begin to refine their decision-making skills and apply their knowledge and abilities to various contexts [5]. To help students build confidence and competence, it is essential to address both threshold concepts (key barriers to knowledge development) and tacit knowledge, particularly within the field of removable prosthetic dentistry. Threshold concepts are fundamental ideas within a field that act as critical barriers or bottlenecks that must be overcome to achieve mastery. Mastering these concepts enhances clinical performance by improving technical accuracy, sharpening practical judgment, and supporting patient-centered decisions, leading to more effective and adaptable treatment in removable prosthodontics (RP) [6-8]. In contrast, tacit knowledge pertains to skills and understanding that are challenging to convey from instructors to learners or between individuals. In the field of RP, proficiency demands not only a grasp of essential concepts, skills, and clinical experience, but also effective patient management.

To help students gain confidence and skills, it is important to focus on both key learning barriers and tacit knowledge. Several factors influence dental students' ability to achieve competency in RP, including the characteristics of learners, instructors, and the learning environment. Experiential learning strengthens RP competency by guiding students through a cycle of doing, thinking, understanding, and refining [9,10]. To address these challenges and the existing knowledge gap, experiential learning has been employed to enhance students’ skills through practical engagement [11]. Experiential learning, commonly used by health professionals, encompasses four stages: concrete experience (active participation in tasks or situations), reflective observation (analysis of the experience), abstract conceptualization (development of personal learning theories), and active experimentation (application of these theories in new contexts) [12].

## Materials and methods

Study design and setting

This study employed an observational, cross-sectional design. Data were collected via a survey conducted from September to June 2023, targeting undergraduate dental students.

Participants

**Figure 1:**
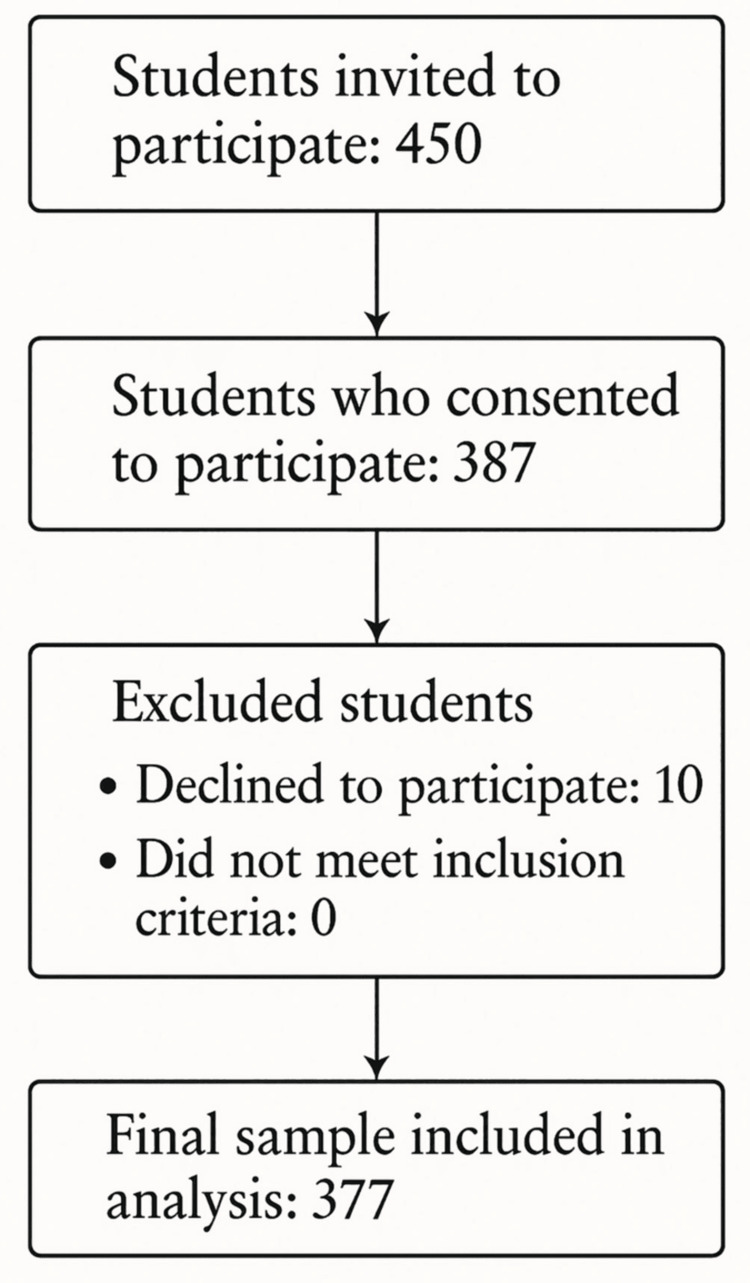
Participant recruitment and selection

Participants were selected using simple random sampling, and 377 dental students who expressed willingness to participate were included in this study. Students were informed of the study's objectives and procedures before their participation. The inclusion criteria encompassed undergraduate dental students from government and private dental schools, specifically clinical students in their third to fifth years who had begun practicing RP. Students who declined to participate in the study were excluded (Figure 1).

Variables

Variables were identified based on questionnaire items developed to explore barriers affecting the comprehension of threshold concepts and the development of cognitive and practical skills related to tacit knowledge. The independent variables consisted of perceived barriers and strategies for addressing these challenges, while the dependent variables reflected participants’ levels of understanding and proficiency in RP.

Data sources/measurement

Data were collected using a structured, self-administered questionnaire developed for this study, based on a comprehensive review of literature on threshold concepts, tacit knowledge, and the development of cognitive and practical skills in RP education. The questionnaire, which included open-ended questions, was distributed both online via Google Forms and offline as printed copies across multiple institutions. The questionnaire consisted of two main sections. The first section collected demographic information, including age, gender, academic background, and level of experience in RP. The second section examined perceived barriers to understanding threshold concepts, focusing on the challenges students face in grasping key ideas and their significance for enhancing oral function and quality of life, as well as evaluating students’ levels of comprehension and proficiency in RP in ways that support improved oral function and overall quality of life.

Students were advised not to take any assistance and respond with their own knowledge. Some responses to open-ended items were subsequently transformed into closed-ended categories for quantitative analysis. To ensure the reliability and validity of the data, all responses were routinely reviewed for clarity, completeness, consistency, and accuracy. Items were measured using a 5-point Likert scale, ranging from 1 (strongly disagree) to 5 (strongly agree). The dependent variables represented participants’ self-assessed comprehension of threshold concepts and proficiency in RP, while the independent variables represented perceived barriers and strategies for overcoming them.

The initial draft of the questionnaire was reviewed by a panel of five experts in medical education, instructional design, and research methodology to ensure content validity and clarity of items. Revisions were made according to their feedback. The revised version was validated with 15 participants who met the inclusion criteria but were not part of the main study sample. Based on their feedback, minor modifications were made to improve item clarity and response consistency. The reliability of the instrument was evaluated using internal consistency analysis. Cronbach’s alpha coefficients were calculated for each construct, with values above 0.70 considered acceptable. The overall questionnaire demonstrated satisfactory reliability across all subscales.

All data were coded and entered into a secure database for analysis. Open-ended responses were analyzed thematically and, where applicable, converted into categorical variables to facilitate statistical testing. Composite scores for each construct were computed by averaging the corresponding item scores. Missing data were handled using pairwise deletion to preserve available information without introducing bias. Data-cleaning procedures were conducted to identify and correct any entry or coding errors before analysis.

Study size

Undergraduate dental students in their third to fifth years at government and private institutions, who had started RP training, were selected via simple random sampling. A total of 377 students who consented to participate were included after being informed about the study’s objectives and procedures, while those who declined were excluded. The sample size was calculated with a 95% confidence level (CI), 5% margin of error, and 50% expected response proportion, ensuring sufficient statistical power.

Statistical methods

Statistical analyses were performed using SPSS Statistics version 23.0 (IBM Corp., Armonk, NY). Descriptive statistics were used to summarize the data and were expressed as percentages for qualitative variables. The Chi-square test was used to analyze relationships within the data. Additional tests, such as the Mann-Whitney U test, Kruskal-Wallis test, and Dwass-Steel-Critchlow-Fligner pairwise comparisons, were applied as necessary to explore specific differences among groups. For the statistical tests, a p-value of <0.05 was considered statistically significant, with a 5% α error and 20% β error, corresponding to a statistical power of 80%.

## Results

This study compared participants’ sex and years of clinical experience in relation to their knowledge of clinical procedures involved in the fabrication of RP. Statistical analysis revealed significant differences in certain areas, whereas other areas showed no significant variation. The comparison of male and female participants, analyzed using the chi-square test, demonstrated that female students significantly outnumbered male students (n = 377, F: 256, M: 121). Furthermore, the data were not normally distributed, so the Mann-Whitney U test revealed a significant difference between male and female participants in the number of complete dentures delivered (M: 32%, F: 68%, p = 0.05) as shown in Figure 1.

However, the Chi-square test indicated no significant gender differences in knowledge related to the steps of complete denture fabrication (p = 0.527). Similarly, no significant gender-based differences were observed in knowledge of anatomical structures, need for assistance during final impression recording, jaw relations, wax try-in, or insertion. Additionally, the male and female participants did not differ significantly in their understanding of dental materials, instruments, or procedures, including opening instruments before starting tasks, occlusal correction, and post-insertion instructions. The need for laboratory technician assistance and knowledge of new materials and techniques also showed no significant sex disparity, except for issues encountered during primary impression making, teeth selection, procedure to clean instruments before working, and packing and curing, where significant differences were observed. The confidence level in the fabrication of RP among genders was evaluated on a scale of 1-10, showing no significant difference between genders (p = 0.55), as shown in Table 1.

**Table 1:** Comparison of responses between female and male undergraduate dental students regarding knowledge and skills related to complete denture fabrication Comparison between gender students regarding the knowledge, skills, problems, and requirements related to the steps in the fabrication of a removable prosthesis. Values are presented as frequency (percentage). The chi-square test (χ²) was used to compare categorical responses between female and male students. For non-parametric continuous data, the Mann-Whitney U test was applied, and for normally distributed continuous data, the Student’s t-test was used. A p-value <0.05 was considered statistically significant NR: no help required; R: required

Question and response	Female, n (%)	Male, n (%)	Test statistic	P-value
Number of complete dentures delivered	—	—	U = 1342	0.051
Knowledge of steps in complete denture fabrication - yes	245 (95.7%)	114 (94.2%)	χ²(1) = 0.400	0.527
Knowledge of steps in complete denture fabrication - no	11 (4.3%)	7 (5.8%)		
Knowledge of related anatomical structures - yes	240 (93.8%)	111 (91.7%)	χ²(1) = 0.750	0.386
Knowledge of related anatomical structures - no	15 (5.9%)	10 (8.3%)		
Need assistance of senior faculty/PGs - yes	244 (95.3%)	115 (95.0%)	χ²(1) = 0.013	0.908
Need assistance of senior faculty/PGs - no	12 (4.7%)	6 (5.0%)		
Help required – primary impression - no help required (NR)	164 (64.1%)	58 (47.9%)	χ²(1) = 8.83	0.003
Help required – primary impression - required (R)	92 (35.9%)	63 (52.1%)		
Help required – final impression - no help required (NR)	94 (36.7%)	43 (35.5%)	χ²(1) = 0.050	0.824
Help required – final impression - required (R)	162 (63.3%)	78 (64.5%)		
Help required – jaw relation - no help required (NR)	151 (59.0%)	64 (52.9%)	χ²(1) = 1.24	0.265
Help required – jaw relation - required (R)	105 (41.0%)	57 (47.1%)		
Help required – wax try-in - no help required (NR)	221 (86.3%)	102 (84.3%)	χ²(1) = 0.276	0.599
Help required – wax try-in - required (R)	35 (13.7%)	19 (15.7%)		
Help required – prosthesis insertion - no help required (NR)	239 (93.4%)	112 (92.6%)	χ²(1) = 0.081	0.775
Help required – prosthesis insertion - required (R)	17 (6.6%)	9 (7.4%)		
Knowledge of dental materials - complete	35 (13.7%)	19 (15.7%)	χ²(1) = 0.276	0.599
Knowledge of dental materials - incomplete	221 (86.3%)	102 (84.3%)		
Material choice based on patient need - based on need	156 (60.9%)	70 (57.9%)	χ²(1) = 0.326	0.568
Material choice based on patient need - based on availability	100 (39.1%)	51 (42.1%)		
Confidence to fabricate a complete denture (1–10 scale)	—	—	t(375) = −0.837	0.403
Knowledge of primary cast material - yes	202 (78.9%)	100 (82.6%)	χ²(1) = 0.721	0.396
Knowledge of primary cast material - no	54 (21.1%)	21 (17.4%)		
Teeth selection knowledge - yes	89 (34.8%)	58 (47.9%)	χ²(1) = 5.99	0.014
Teeth selection knowledge - no	167 (65.2%)	63 (52.1%)		
Knowledge of packing and curing - complete	25 (9.8%)	28 (23.1%)	χ²(1) = 12.57	<0.001
Knowledge of packing and curing - incomplete	231 (90.2%)	93 (76.9%)		
Instrument use knowledge - complete	12 (4.7%)	5 (4.1%)	χ²(1) = 0.059	0.808
Instrument use knowledge - incomplete	244 (95.3%)	116 (95.9%)		
Need for laboratory assistance - every time	111 (43.4%)	42 (34.7%)	χ²(2) = 3.31	0.191
Need for laboratory assistance - few stages	118 (46.1%)	62 (51.2%)		
Need for laboratory assistance - none	25 (9.8%)	17 (14.0%)		
Knowledge of new materials/techniques - yes	65 (25.4%)	25 (20.7%)	χ²(1) = 1.01	0.315
Knowledge of new materials/techniques - no	191 (74.6%)	96 (79.3%)		
Correct opening of instruments - correct	98 (38.3%)	53 (43.8%)	χ²(1) = 1.04	0.307
Correct opening of instruments - incorrect	158 (61.7%)	68 (56.2%)		
Knowledge of instrument cleaning - complete	16 (6.2%)	1 (0.8%)	χ²(1) = 5.61	0.018
Knowledge of instrument cleaning - incomplete	240 (93.8%)	120 (99.2%)		
Recommendation – demonstrations - yes	77 (30.1%)	30 (24.8%)	χ²(1) = 1.13	0.288
Recommendation – demonstrations - no	179 (69.9%)	91 (75.2%)		
Recommendation – increase practice hours - yes	62 (24.2%)	32 (26.4%)	χ²(1) = 0.218	0.641
Recommendation – increase practice hours - no	194 (75.8%)	89 (73.6%)		
Recommendation – teacher assistance - yes	50 (19.5%)	26 (21.5%)	χ²(1) = 0.150	0.698
Recommendation – teacher assistance - no	203 (79.3%)	95 (78.5%)		
Recommendation – others - yes	87 (34.0%)	38 (31.4%)	χ²(1) = 0.247	0.619
Recommendation – others - no	169 (66.0%)	83 (68.6%)		
Post-insertion instructions knowledge - adequate	21 (8.2%)	9 (7.4%)	χ²(1) = 0.066	0.798
Post-insertion instructions knowledge - inadequate	235 (91.8%)	112 (92.6%)		

**Figure 2:**
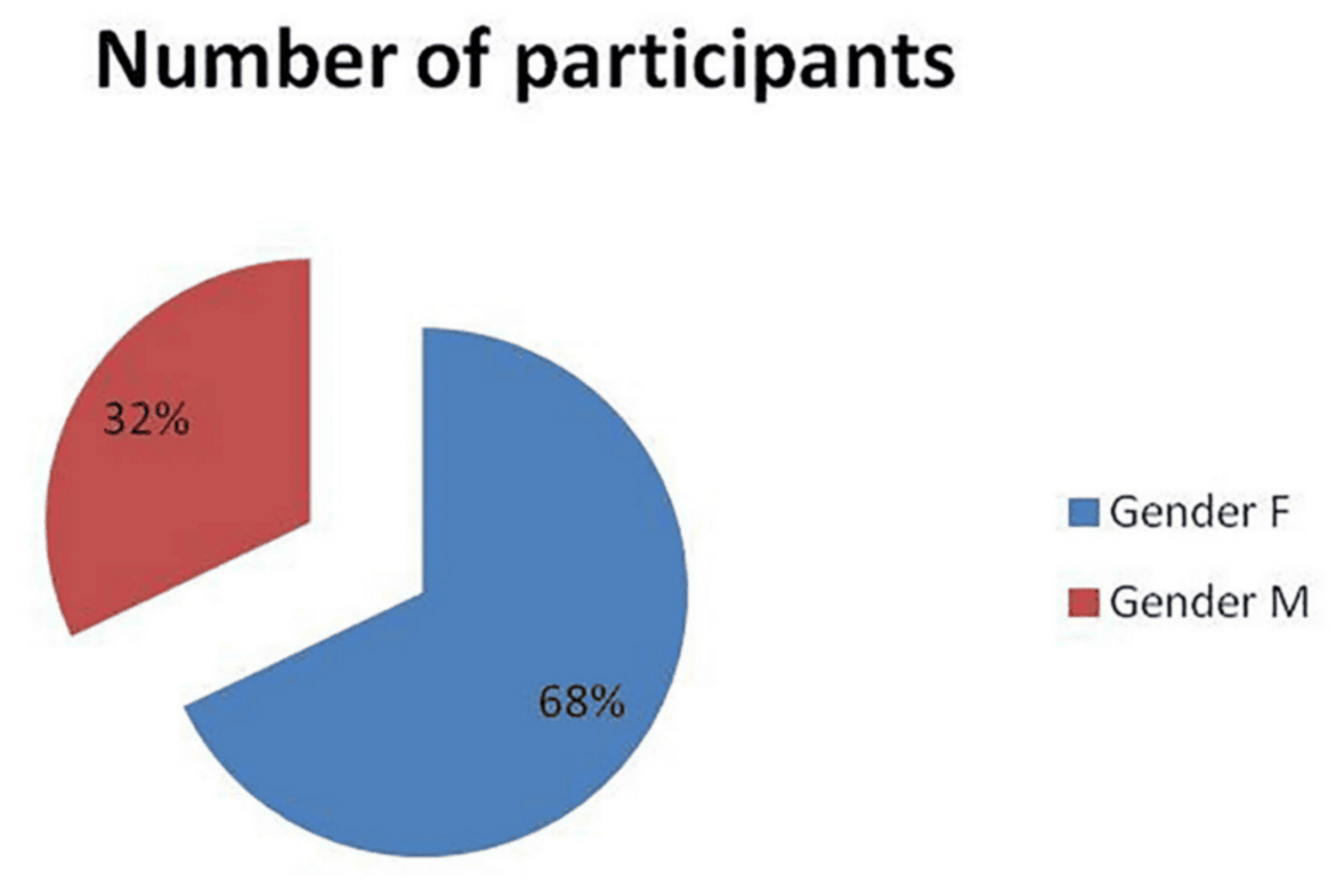
Total participants by gender Gender distribution among the study participants (N = 377). A total of 68% of participants were female, and 32% were male. Values are presented as descriptive percentages of the overall sample

Participants from three years of clinical study were compared in terms of their skills and knowledge (third year: 28%, fourth year: 54%, fifth year: 18%), as shown in Figure 2. When comparing students from different clinical years, the chi-square test revealed significant differences in knowledge of steps for complete denture fabrication, anatomical structures, the use of dental materials based on patient needs or store availability, display of the instruments before procedure, and during the occlusal corrections procedure (p = 0.005, p = 0.003, p = 0.001, p = 0.006 and p<0.001 respectively). Differences were also evident in the awareness of new materials and techniques, procedural readiness, and teacher assistance. However, there were no significant differences among students regarding the help needed for primary impressions, jaw relations, try-in, insertion, confidence in RP fabrication, or understanding of dental materials and instruments. The challenges faced during the packing and curing of RP also showed no significant variation across the years (p = 0.987), as presented in Table 2.

**Table 2:** Comparison of knowledge, skills, challenges, and support requirements among students across different study years in the fabrication of removable prostheses Comparison of students in different study years regarding their knowledge, skills, problems, and requirements related to the steps in the fabrication of a removable prosthesis. "KW χ²" and "χ²" represent Kruskal-Wallis and Chi-Square tests, with "df" indicating degrees of freedom. For pairwise comparisons, the Dwass-Steel-Critchlow-Fligner (DSCF) method was used. A p-value <0.05 was considered statistically significant N/A: not applicable

Question	Response level	Year 3. n (%)	Year 4, n (%)	Year 5, n (%)	Statistic (p-value)
How many complete dentures have you delivered so far?	—	N/A	N/A	N/A	KW χ² = 45.4 (2), p<0.001
					Pairwise (DSCF):
					3 vs 4: W = 2.94, p = 0.094
					3 vs 5: W = 8.11, p<0.001
					4 vs 5: W = 8.66, p<0.001
Do you know all the steps in the fabrication of a complete denture? (1 = yes; 2 = no)	Yes	94 (89.5)	198 (97.5)	67 (97.1)	χ² = 10.43 (2), p = 0.005
	No	11 (10.5)	5 (2.5)	2 (2.9)	
Knowledge of anatomical structures associated with fabrication? (1 = yes; 2 = no)	Yes	91 (86.7)	192 (95.0)	68 (98.6)	χ² = 11.50 (2), p = 0.003
	No	14 (13.3)	10 (5.0)	1 (1.4)	
Ability to work independently (1 = need help; 2 = help not needed)	Need help	99 (94.3)	198 (97.5)	62 (89.9)	χ² = 6.97 (2), p = 0.031
	Help not needed	6 (5.7)	5 (2.5)	7 (10.1)	
Stage where maximum help is needed (0 = not required; 1 = required)	Primary	47 (44.8)	87 (42.9)	21 (30.4)	χ² = 4.08 (2), p = 0.130
	Final	60 (57.1)	143 (70.4)	37 (53.6)	χ² = 8.97 (2), p = 0.011
	Jaw	48 (45.7)	81 (39.9)	33 (47.8)	χ² = 1.77 (2), p = 0.413
	Wax	15 (14.3)	33 (16.3)	6 (8.7)	χ² = 2.40 (2), p = 0.301
	Insert	8 (7.6)	14 (6.9)	4 (5.8)	χ² = 0.22 (2), p = 0.898
Do you have complete knowledge of the materials used in removable prosthesis? (1 = complete; 2 = incomplete; 3 = none)	Complete	14 (13.3)	29 (14.3)	11 (15.9)	χ² = 0.23 (2), p = 0.891
	Incomplete	91 (86.7)	174 (85.7)	58 (84.1)	
	No knowledge	0 (0.0)	0 (0.0)	0 (0.0)	
Do you use materials according to the patient's needs or availability? (1 = according to need; 2 = whatever available)	According to need	47 (44.8)	139 (68.5)	40 (58.0)	χ² = 16.34 (2), p<0.001
	Whatever available	58 (55.2)	64 (31.5)	29 (42.0)	
Knowledge of primary cast material (1 = yes; 2 = no)	Knowledge	87 (82.9)	158 (77.8)	57 (82.6)	χ² = 1.44 (2), p = 0.486
	No knowledge	18 (17.1)	45 (22.2)	12 (17.4)	
Knowledge of teeth selection (1 = yes; 2 = no)	Knowledge	12 (11.4)	98 (48.3)	37 (53.6)	χ² = 44.03 (2), p<0.001
	No knowledge	93 (88.6)	105 (51.7)	32 (46.4)	
Knowledge of packing and curing cycle (1 = complete; 2 = incomplete)	Complete	15 (14.3)	28 (13.8)	10 (14.5)	χ² = 0.03 (2), p = 0.985
	Incomplete	90 (85.7)	175 (86.2)	59 (85.5)	
Instrument use as per procedure (1 = complete; 2 = incomplete)	Complete	3 (2.9)	11 (5.4)	3 (4.3)	χ² = 1.06 (2), p = 0.588
	Incomplete	102 (97.1)	192 (94.6)	66 (95.7)	
Need for laboratory technical assistance (1 = every; 2 = few; 3 = none)	Every case	53 (50.5)	75 (36.9)	25 (36.2)	χ² = 8.20 (4), p = 0.085
	Few cases	40 (38.1)	103 (50.7)	39 (56.5)	
	Not required	12 (11.4)	25 (12.3)	5 (7.2)	
Confidence to fabricate a denture (scale 1–10)	Distribution provided	—	—	—	t = -0.837 (375), p = 0.403; mean diff = -0.137, SE diff = 0.164 95% CI: [-0.460, 0.185]
Do you have knowledge of new materials and techniques? (1 = no; 2 = yes)	No	83 (79.0)	164 (80.8)	40 (58.0)	χ² = 13.74 (2), p = 0.001
	Yes	22 (21.0)	39 (19.2)	29 (42.0)	
Opening instruments in front of patient (1 = correct; 2 = incorrect)	Correct	40 (38.1)	94 (46.3)	17 (24.6)	χ² = 9.67 (2), p = 0.008
	Incorrect	65 (61.9)	109 (53.7)	52 (75.4)	
Instrument cleaning before working (1 = complete; 2 = incomplete)	Complete	6 (5.7)	9 (4.4)	2 (2.9)	χ² = 0.76 (2), p = 0.684
	Incomplete	99 (94.3)	194 (95.6)	67 (97.1)	
Recommendations to improve teaching or skills	Demo	36 (34.3)	58 (28.6)	13 (18.8)	χ² = 15.94 (6), p = 0.014
	More hours	20 (19.0)	50 (24.6)	24 (34.8)	
	More teachers	30 (28.6)	38 (18.7)	8 (11.6)	
	Others	28 (26.7)	72 (35.5)	25 (36.2)	
Knowledge of occlusal correction (0 = no; 1 = adequate; 2 = inadequate)	No knowledge	18 (17.1)	15 (7.4)	1 (1.4)	χ² = 34.05 (4), p<0.001
	Adequate	0 (0.0)	5 (2.5)	9 (13.0)	
	Inadequate	87 (82.9)	183 (90.1)	59 (85.5)	
Post-insertion instructions given (1 = adequate; 2 = inadequate)	Adequate	13 (12.4)	12 (5.9)	5 (7.2)	χ² = 4.04 (2), p = 0.132
	Inadequate	92 (87.6)	191 (94.1)	64 (92.8)	

**Figure 3:**
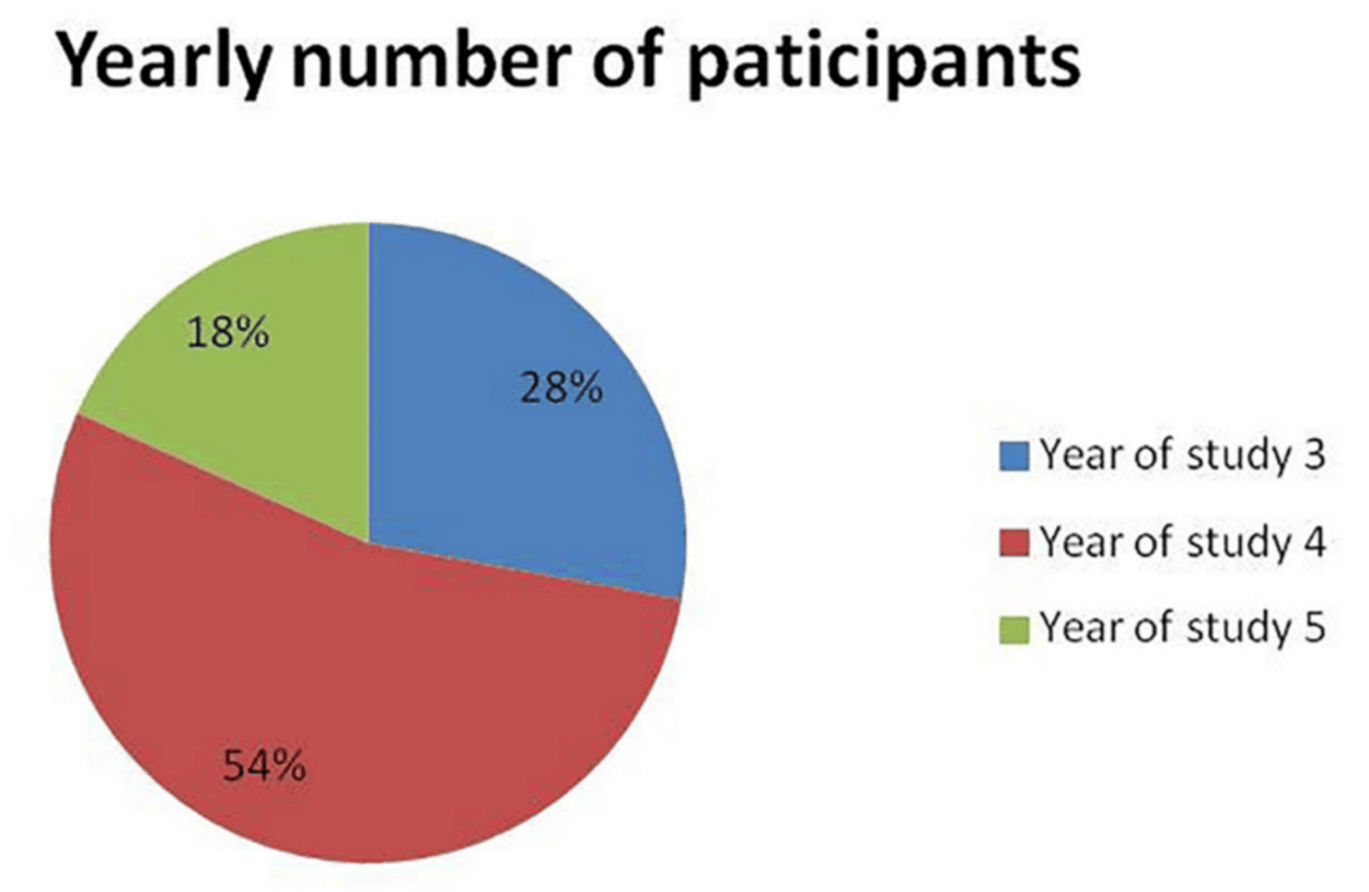
Total participants by study year Distribution of participants by year of study (N = 377): 28% were in the third year, 54% in the fourth year, and 18% in the fifth year. Values are presented as descriptive percentages

The Kruskal-Wallis test was used to assess the number of complete dentures delivered by students from different clinical years (mean values for third year: 3.44, fourth year: 3.9, fifth year: 6.88, p<0.01) as shown in Figure 3. Significant differences were identified among the clinical groups, with pairwise comparisons using the Dwass-Steel-Critchlow-Fligner method, revealing notable differences between third- and fifth-year students as well as between fourth- and fifth-year students (p<0.01), as shown in Table 2.

Table 4 shows the mean number of dentures made by students from different academic years.

**Figure 4:**
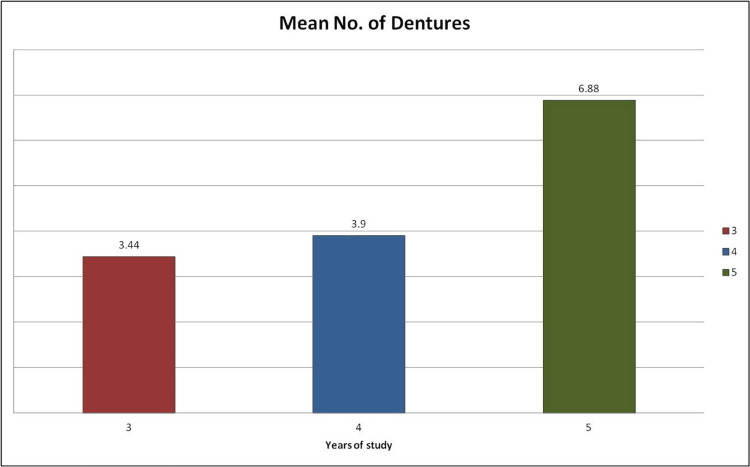
Mean number of dentures made by students from different academic years Mean number of dentures completed by students across years of study. The mean number of dentures completed was 3.44 in the third year, 3.9 in the fourth year, and 6.88 in the fifth year. Values are presented as descriptive statistics (means)

## Discussion

This study investigates key challenges in learning RP, focusing on threshold concepts and the development of tactile knowledge. It highlights the importance of students developing both theoretical knowledge and psychomotor skills and successfully integrating them into clinical practice to achieve RP competency. The research also explores strategies to address learning obstacles by considering both intrinsic factors (e.g., personal motivation, self-discipline) and extrinsic factors (e.g., teaching methods, learning environment), alongside experiential learning. Rather than relying solely on exam scores or grades, students’ understanding and challenges were assessed through interviews, providing deeper insights into their learning experiences.

Deep learning involves connecting different pieces of knowledge, understanding information on a deeper level, and linking concepts together [13,14]. In RP, students must understand oral tissues and the movement and function of denture components, yet they often struggle to apply this knowledge to real-life clinical situations or individual patients. Additionally, when too much information is presented in a short time, students may feel overwhelmed, making it harder for them to progress in their learning. This situation aligns with the cognitive load theory, which highlights the limitations of human working memory [15,16].

The present study compared the knowledge and competencies of students across three clinical years regarding RP fabrication, with statistical analysis conducted using the chi-square test. The findings indicated significant differences among the students in various aspects of complete denture fabrication. Notably, variations were observed in their understanding of procedural steps, anatomical structures, and the need for assistance. Additionally, differences were found in the students' ability to select dental materials based on patient needs or store availability, familiarity with new materials and techniques, awareness of proper instrument handling before initiating procedures, and the extent of guidance received from instructors.

These findings suggest that students’ theoretical knowledge and practical skills developed at different rates across the three clinical years. Chi-square analysis of interview data demonstrated significant improvements with advancing year in areas such as procedural understanding, anatomy, material selection, technique familiarity, and reduced reliance on assistance, likely driven by increased clinical exposure and enhanced instructional support. However, basic clinical steps (e.g., impressions, jaw relations, try-ins), material knowledge, confidence, and packing/curing challenges showed no significant change. These findings highlight the need for targeted teaching interventions to achieve balanced competency. Conversely, no statistically significant differences were observed for certain domains, including the need for assistance in making primary impressions, establishing jaw relationships, conducting try-ins, and completing insertions. Moreover, the students demonstrated similar levels of knowledge regarding dental materials, material selection for pouring primary casts, instrument identification, and overall confidence in RP fabrication. Additionally, the challenges faced during the packing and curing phases of prosthesis fabrication were consistent across the different years of study.

These results indicate that, while certain aspects of prosthodontic training improve significantly over time, others remain relatively unchanged. To more appropriately address the study’s objective, the main challenges identified - cognitive overload, varying levels of required assistance, and continued difficulties with procedures like packing and curing - can be addressed through targeted improvements. Introducing hands-on simulation with repetitive practice would ease cognitive load and strengthen tacit knowledge. Implementing mentorship with structured, feedback-driven debriefs would help link errors to theoretical concepts and reduce inconsistencies in support needs. Incorporating peer discussion groups would encourage collaborative problem-solving. Finally, hybrid experiential modules could directly target non-progressing skill domains and have shown evidence of improving procedural confidence and performance.

Many students struggle with RP because they do not clearly see how classroom knowledge connects to real patient care. Even though they learn about oral tissues and how dentures work, applying this information to actual cases is often difficult. Owing to the intricacies of threshold concepts, some students may get stuck within the liminal state, lacking scaffolds or essential frameworks to assimilate new information based on their existing knowledge [7-9].

Limitations of the study

This study has certain limitations. Its cross-sectional design offers only a snapshot of students’ knowledge and skills, and the reliance on self-reported data may introduce response bias. Furthermore, the sample had an uneven gender distribution, with more females than males, and the three dental year groups were not equally represented, which may limit the generalizability of the findings. Future research should adopt a longitudinal approach to monitor students’ competency development over time, include postgraduate participants for broader comparison, and evaluate the effectiveness of targeted educational strategies such as workshops, mentorship programs, and enhanced integration of theoretical and clinical training to strengthen learning outcomes in RP.

## Conclusions

This study aims to enhance dental educators' understanding of the educational context and support improvements in teaching and learning in undergraduate dental curricula. To build on the findings presented, future research should explore the distinctive aspects that distinguish undergraduate dental education from other health professions and examine the roles and contributions of key stakeholders, such as students and educators. Ultimately, the study seeks to serve as a foundation and inspiration for the continued advancement of the dental profession.

## Appendices

**Table 3:** Questionnaire Survey: Difficulties Encountered in Removable Prosthodontics by Undergraduates

Section	Response	
Email		
Name		
Year		
College Name		
Question	Response	Options / Notes
1. How many complete dentures have you delivered so far?		
2. Do you know all the steps in fabrication of complete denture?		☐ Yes ☐ No
3. At which stage are you most confident?		☐ Primary impression ☐ Final impression ☐ Jaw relation ☐ Try-in stage ☐ Insertion and follow-up
4. What impression materials do you commonly use?		☐ Alginate ☐ Impression compound ☐ Elastomeric impression materials ☐ Denture base resins
5. Do you have knowledge of the anatomical structures associated with fabrication of complete denture?		☐ Yes ☐ No
6. Can you evaluate and work on a patient alone or do you need help from senior faculty/postgraduates?		
7. At what stage do you need maximum help?		
8. Do you have complete knowledge of dental materials used in removable prosthesis?		☐ Yes ☐ No
9. Do you use materials according to the patient’s need or based on availability in the college?		
10. In which material do you pour the primary cast?		☐ Dental plaster ☐ Dental stone ☐ Die stone
11. How do you do the teeth selection for the patient?		
12. What problems do you face while packing and curing cycle?		
13. What instruments do you use during fabrication of removable prosthesis?		
14. In how many cases do you need laboratory technical assistance?		
15. How confident are you to fabricate a complete denture in a clinical setup?		1 = Not confident 10 = Very confident
16. Do you know about new materials and techniques in complete denture fabrication?		☐ Yes ☐ No
17. How do you open instruments in front of the patient?		
18. What steps do you take to clean instruments before working on a patient?		
19. What recommendations would you introduce to improve teaching or your working skills?		
20. When is the occlusal correction done?		☐ Immediately ☐ During insertion ☐ One week after insertion
21. What post-insertion instructions are given to the patient?		
